# Diagnosis and treatment of isolated snoring—open questions and areas for future research

**DOI:** 10.1007/s11325-020-02138-6

**Published:** 2020-07-04

**Authors:** B. Hofauer, B. Braumann, C. Heiser, M. Herzog, J. T. Maurer, S. Plößl, J. U. Sommer, A. Steffen, T. Verse, B. A. Stuck

**Affiliations:** 1 Department of Otorhinolaryngology/Head and Neck Surgery, University Medical Center Freiburg, Killianstr. 5, 79106 Freiburg, Germany; 2 Department of Maxillofacial Surgery/Orthodontics, University of Cologne, Cologne, Germany; 3 Department of Otorhinolaryngology/Head and Neck Surgery, Klinikum rechts der Isar, Technical University Munich, Munich, Germany; 4 Department of Otorhinolaryngology/Head and Neck Surgery, Carl-Thiem-Hospital Cottbus, Cottbus, Germany; 5 Department of Otorhinolaryngology/Head and Neck Surgery, University Hospital Mannheim, Mannheim, Germany; 6 Department of Otorhinolaryngology/Head and Neck Surgery, University Hospital Halle, Halle, Germany; 7 Department of Otorhinolaryngology/Head and Neck Surgery, Helios University Hospital Wuppertal, Wuppertal, Germany; 8 Department of Otorhinolaryngology/Head and Neck Surgery, University Hospital Schleswig-Holstein, Lübeck, Germany; 9 Department of Otorhinolaryngology/Head and Neck Surgery, Asklepios Hospital Hamburg Harburg, Hamburg, Germany; 10 Department of Otorhinolaryngology/Head and Neck Surgery, University Hospital Marburg, Philipps-Universit Marburg, Marburg, Germany

**Keywords:** Snoring, Diagnosis, Therapy, Review, Adults

## Abstract

**Study objectives:**

Snoring is a common phenomenon which is generated by vibration of soft tissue of the upper airway during sleep. Due to the high incidence of isolated snoring and the substantial burden for the patient and the bed partner, a thorough examination and appropriate therapy are required. Many recommendations for the treatment of isolated snoring are either not evidence-based or are derived from recommendations for the management of obstructive sleep apnea. Therefore, the aim of this study is the identification and description of open questions in the diagnosis and treatment of isolated snoring and the illustration of areas for further research.

**Methods:**

In the context of the development of the new version of the German guideline “Diagnosis and treatment of isolated snoring in adults,” a multidisciplinary team of experts performed a systematic literature search on the relevant medical data and rated the current evidence regarding the key diagnostic and therapeutic measures for snoring.

**Results:**

The systematic literature review identified 2293 articles. As a major inclusion criterion, only studies on primary snoring based on objective sleep medical assessment were selected. After screening and evaluation, 33 full-text articles remained for further analysis. Based on these articles, open questions and areas for future research were identified for this review.

**Conclusion:**

Several major gaps in the literature on the diagnosis and treatment of isolated snoring were identified. For the majority of diagnostic and therapeutic measures for snoring, high-level scientific evidence is still lacking.

## Introduction

Snoring as an isolated phenomenon is very common. The symptom is generated by vibration of the soft tissue of the upper airway during sleep. According to the International Classification of Sleep Disorders (ICSD-3), isolated snoring is categorized under sleep-related breathing disorders, but must be differentiated from obstructive sleep apnea, either by home sleep testing (HST) or polysomnography (PSG) [1, 2]. According to the National Sleep Foundation, 90 million Americans report isolated snoring, of which 37 million declare to snore routinely [3]. In general, epidemiological investigations on the prevalence of isolated snoring are difficult due to the fact that the necessary differentiation from snoring as part of obstructive sleep apnea (OSA) is not routinely performed. Additionally, a universal definition of this acoustic phenomenon is not available and therefore the widely reported incidence ranges from 2 to 86% [4, 5]. Standardized telephone interviews resulted in a frequency of snoring of 20% in women and 26% in men up to the age of 24 years with the highest frequency between 45 and 55 years [6]. It is proven that snoring is more frequent in men than in women [7]. Besides gender and age, elevated body weight, impaired nasal passage, velar hyperplasia, smoking, and alcohol consumption are further risk factors.

Due to the high incidence of snoring and the substantial burden for the patient and the bed partner, a thorough examination and appropriate therapy for snoring are required. It is still part of ongoing discussion and investigation, whether or not isolated snoring is a cardiovascular risk factor. In a comparison of 377 subjects with isolated snoring after PSG exclusion of OSA with 264 healthy subjects, no difference regarding fatal and non-fatal cardiovascular events “could be detected after adjustment for the common risk factors [8]. However, Lee et al. observed a correlation between arteriosclerosis of the carotid artery and snoring intensity in a collection of 110 subjects with isolated snoring and excluded OSA via PSG [9].

The role of isolated snoring as potential risk factor for cardiovascular diseases remains unclear—also in the diagnosis and especially in the treatment of snoring, many recommendations are either not evidence-based or derived on recommendations for the management of patients with OSA, despite the fact that snoring in the context of OSA is not equivalent to snoring as an isolated phenomenon. Therefore, the discrimination between isolated snoring and snoring as part of OSA is essential in order to indicate the appropriate treatment. Thus, the aim of this review is the identification and description of current gaps in the evidence of diagnosis and treatment of isolated snoring and the illustration of areas for further research.

## Material and methods

In the context of developing the new version of the German S3 guideline on “diagnosis and therapy of snoring in adults,” a multidisciplinary team of ten experts defined multiple topics in the management of snoring, for which the evidence should be evaluated [10, 11]. The panel contained members with extensive clinical and scientific experience in the fields of otorhinolaryngology/head and neck surgery, maxillofacial surgery, and sleep medicine. The following study questions were defined:

### Diagnosis of isolated snoring

- Is the application of drug-induced sleep endoscopy (DISE) beneficial regarding the therapeutic outcome?
- Is the application of pressure catheter beneficial regarding the therapeutic outcome?

### Therapy of isolated snoring

- What is the evidence for the effectiveness of positional treatment of isolated snoring?
- What is the evidence for the effectiveness of myofunctional treatment of isolated snoring?
- What is the evidence for the effectiveness of weight loss in the treatment of isolated snoring?
- What is the evidence for the effectiveness of a treatment with a mandibular advancement device (MAD) in isolated snoring?
- Is the application of individual MAD superior compared to ready-for-use MAD in the treatment of isolated snoring?
- Does the effectiveness of MAD in the treatment of isolated snoring depend on the amount of advancement/protrusion?
- What is the evidence for the effectiveness of soft palate surgery in the treatment of isolated snoring?
- What is the evidence for the effectiveness of nasal surgery in the treatment of isolated snoring?

### Systematic literature research

In January 2018, a literature search was conducted for existing Cochrane reviews in the Cochrane library under the term of “snoring” without date restriction. Additionally, a literature research for existing guidelines or systematic reviews under the term of “snoring” and the limits of “guideline,” “systematic reviews,” “human,” “adults,” “English,” and “German” was performed. In February 2018, a systematic literature research by a certified librarian (Maurizio Grilli, MLIS, Library of the Medical Faculty of Mannheim, University of Heidelberg, Germany) in the databases of PubMed, Cochrane Library, Web of Science Core Collection, and ClinicalTrial.gov, starting from the year 2000, has been conducted. This time, restriction has been chosen since a differentiation between isolated snoring and OSA has not been done consequently in earlier publications.

### Review of the literature

The results from the systematic literature research have been thematically separated and allocated to the authors. In the first step, all abstracts were screened by at least two authors and excluded if obviously irrelevant for the single topics. Abstracts were included, if at least one author defined them as relevant. In the second step, all relevant abstracts were reevaluated on the basis of the full-texts and were either included, if again at least one of two authors defined the articles as relevant, or excluded, if still irrelevant for the single topics. At this stage, the reason for exclusion was documented. The selection was limited to English or German articles, adult patients in whom OSA was objectively excluded (AHI < 5/h) and, in the case of articles evaluating therapeutic modalities, to studies with at least ten patients. The information flow through the different phases of the systematic review is depicted as flow diagram according to the PRISMA recommendations [12]. The articles that were finally identified were evaluated independently by two authors and evaluated according to Oxford Level of Evidence (https://www.cebm.net/2009/06/oxford-centre-evidence-based-medicine-levels-evidence-march-2009/).

### Identification of current gaps in the literature

Questions on the diagnosis and treatment of isolated snoring, which could not be answered by the existing literature, were identified and described as current gaps and areas for future research.

## Results

### Systematic review of the literature

**Fig. 1:**
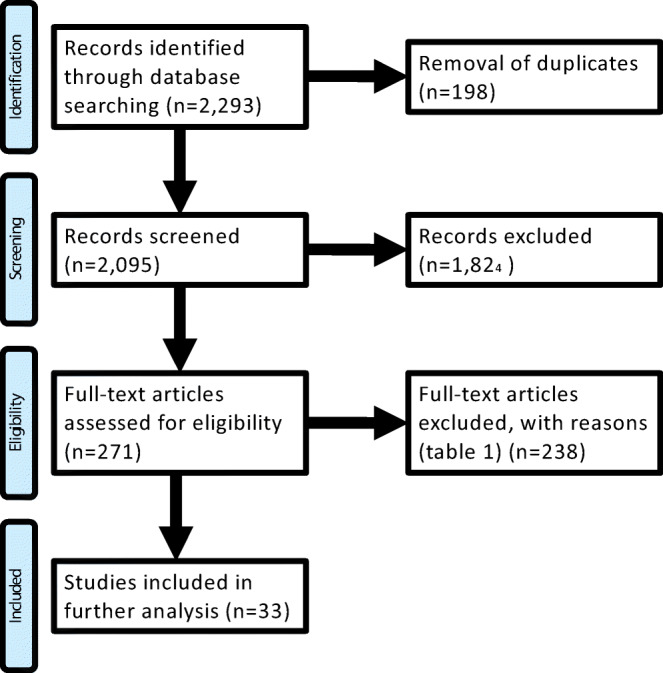
PRIMSA to illustrate the flow of information through the different phases of the systematic review

**Fig. 2:**
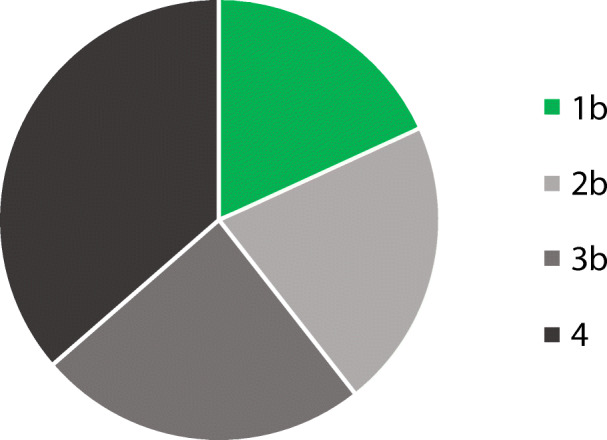
Illustration of the distribution of the levels of evidence of the existing literature in the diagnosis and treatment of snoring

No existing Cochrane reviews on isolated snoring could be identified. A clinical practice guideline of the American Academy of Sleep Medicine and the American Academy of Dental Sleep Medicine on the application of oral appliances could be identified and was included in the particular statement [13]. Two systematic reviews on relevant topics of this guideline could be identified and were evaluated based on standardized criteria (revised Assessment of Multiple SysTemAtic Reviews (AMSTAR)) and were included accordingly [14–16]. The systematic review of the literature resulted in 2293 articles. A total of 2095 abstracts of these identified articles were screened, of which 1824 abstracts were excluded as not relevant for the particular topic. In a next step, 271 full-texts were evaluated by the authors and subsequently 238 full-texts were excluded with documentation of the exclusion reason (Table 1). The most frequent reason for exclusion was the insufficient exclusion of OSA; other reasons are illustrated in Table 1. Finally, 33 articles were defined for the final development of the recommendations (Fig. 1); the levels of evidence are illustrated in Fig. 2. All articles are cited within the German S3 guideline on “diagnosis and therapy of snoring in adults” [10].

**Table 1:** Reasons for the exclusion of the full-text articles

Full-text article was not available	18
Full-text article not in German or English language	4
OSA no definitely excluded	137
Patient number < 10	8
Format of full-text article	5
Non-systematic review	6
Other reasons for exclusion	60

### Diagnosis of isolated snoring

DISE allows the identification of the origin of snoring sounds and the differentiation from OSA, but pharyngeal pressure catheters have not been under evaluation in patients with isolated snoring [17]. The raised questions on the potential benefit of the application of DISE and pressure catheters could not be answered with the available literature, since no sufficient evidence was available based on the abovementioned criteria for the selection of clinical studies.

### Therapy of isolated snoring

No evidence was available for the effect of positional therapy in the treatment of isolated snoring in adults, since all screened abstracts and full-texts on this topic were excluded mainly due to imprecise in- or exclusion criteria (e.g., OSA not definitely excluded). The only evidence on positional therapy for isolated snoring was available on positional therapy of the head, which is reported to be effective in the subjective and objective intensity of snoring (evidence level 1b) [18]. In a current systematic review on positional OSA, the effect on isolated snoring was evaluated [19]. The reported results were inhomogeneous in this review and it was stated that isolated positional snoring is uncommon. Studies on isolated snoring were again not reported.

The literature research on myofunctional therapy for isolated snoring in adult patients did not reveal any evidence for or against the recommendation of this treatment.

Studies evaluating the effect of weight loss on snoring intensity in patients with isolated snoring without OSA have not been conducted so far. Since body weight is a risk factor of snoring, therapeutic efficacy may be presumed, although this has not been demonstrated to date. This is of particular interest, since weight reduction is a standard recommendation in subjects with isolated snoring (evidence level 5).

In contrast, with placebo-controlled trials, evidence on the effectiveness of a treatment with a MAD in adults with isolated snoring was available and further studies also demonstrated the beneficial application of MAD for this indication (evidence level 1b) [20–23]. One placebo-controlled study on the effect of a thermoplastic MAD was available that fulfilled the inclusion criteria, and therefore, the recommendation includes custom-made and thermoplastic MAD [24]. The effective reduction of both objective (snores per hour, detected during home sleep test) and subjective snoring intensity (Snoring Symptoms Inventory, sleeping partner snore VAS) could be demonstrated. However, a direct comparison of custom-made and thermoplastic MAD in patients with isolated snoring has not been conducted yet. Data on long-term effectiveness and side effects regarding treatment with thermoplastic MAD specifically for isolated snoring were not available. The correlation of the effectiveness of MAD therapy with the amount of advancement respectively, protrusion, as well as potential clinical predictors for the effectiveness of MAD therapy could not be answered with the available literature. Further research in this area should be conducted, especially considering the fact that MAD devices for patients with isolated snoring are usually not covered by health insurance.

The available literature provided limited evidence on the effect of nasal surgery in patients with impaired nasal breathing on the intensity of snoring. Four case-control studies have been identified, which evaluated the effect of nasal surgery on the subjective and objective snoring intensity (evidence level 3b) [25–28]. In all studies, the subjective snoring intensity was improved by nasal surgery, but this effect could not be demonstrated with objective endpoints and it could further be shown that palate or mandibular surgery reduced the snoring intensity more effectively. There is no evidence, however, on the effect of nasal surgery on the snoring intensity of patients without subjective impairment of nasal breathing.

With 16 publications meeting the inclusion criteria, evidence on the effectiveness of soft palate surgery in the treatment of isolated snoring in adults was available. Different surgical approaches have been under investigation: Uvulopalatopharyngoplasty, uvulopalatoplasty, modifications such as radiofrequency-assisted or laser-assisted uvulopalatoplasty, radiofrequency therapy of the palate and palatal implants. Due to a randomized, placebo-controlled trial, the highest level of evidence is available for radiofrequency therapy of the soft palate (evidence level 1b) [29]. Comparing the insertion of the radiofrequency needle into the soft palate without further application of energy to regular radiofrequency therapy, a significantly different effect in favor of radiofrequency therapy has been observed. While combined radiofrequency-assisted uvulopalatoplasty increased the observed efficacy, the snoring intensity gradually increased during long-term follow-up [30, 31]. Further evidence was available for the application of soft palate implants, representing another minimally invasive procedure for the treatment of isolated snoring (evidence level 4) [32]. Other surgical interventions, such as maxillomandibular advancement or surgery of the larynx or hypopharynx, have not been or have not been adequately checked in the therapy of isolated snoring and there is insufficient data for this indication. In addition, the invasiveness of many procedures appears to be problematic when it comes to snoring.

The identified gaps in the literature regarding diagnosis and treatment of isolated snoring in adults and possible areas for future research are summarized in Table 2.

**Table 2:** Possible areas for future research in the diagnosis and treatment of snoring in adults

− What is the additional predictive value of DISE and pharyngeal pressure	
− Catheters regarding the choice of treatment in snoring?	
− How effective is positional therapy in patients with isolated snoring?	
− How effective is weight loss in overweight patients with isolated snoring?	
− What are clinical predictors for the efficacy of MAD therapy in patients with isolated snoring?	
− What comorbidities profit from the therapy of isolated snoring?	

## Discussion

As part of the development of the current version of the German guideline on the diagnosis and treatment of isolated snoring in adults and the associated systematic literature research, striking and partially unexpected gaps in the related literature were identified. This review was conducted in order to illustrate the areas of missing evidence, both in diagnosis and treatment of this common disorder, and thereby stimulate prospective clinical trials.

Although in the first round of the evaluation many abstracts were identified, screened and numerous full-texts were evaluated, only few studies met the inclusion criteria of this evaluation. The most frequent reason for exclusion of many articles was the insufficient differentiation between isolated snoring and snoring as part of OSA. Although a number of studies performed a home sleep test or polysomnography to rule out OSA, cutoff values that did not sufficiently differentiate isolated snoring from OSA were frequently used, which led to the inclusion of patients with snoring in combination with mild to moderate sleep apnea. Therefore, it is highly recommended, in accordance with the ICSD-3, to conduct either a home sleep test or polysomnography with an AHI of < 5 events per hour as cutoff at baseline as inclusion criterion both for diagnostic and therapeutic studies in isolated snoring [1].

The multidisciplinary working group further defined relevant outcome parameters which should receive special attention (the ranking corresponds to the importance of the parameter):

- Subjective snoring intensity including subjective scores
- Unwanted effects/morbidity
- Quality of life
- Acoustic snoring analysis including objective methods of investigation and classification
- Costs of the procedure

With regard to the most important outcome parameter, the subjective snoring intensity, a particular characteristic, has to be taken into account. Usually, the primary outcome parameters are patient-relevant endpoints and therefore in the focus of guidelines or the evaluation of the effectiveness of diagnostic or therapeutic procedures. In isolated snoring, however, the subjective evaluation of the snoring intensity is not evaluated by the patients for obvious reasons, but by the bed partners. The affected individuals usually do not suffer from their own snoring, but from the extent of the annoyance of the environment. This justifies why, in the present case, the subjective harassment of the bed partners was selected as the most important endpoint, as it is also customary in the international context. However, evaluation with subjective scores is prone to bias and only the reflection of the current bed partner. The authors around De Meyer et al. demand the development of a model for assessing snoring, which includes both properties of the sound, but also physiological aspects, such as the annoyance influenced by personality aspects, sensitivity to noise, and environmental factors [5].

Visual analogue scales offer the ability to classify snoring by volume and frequency. Similar to questionnaires, such as the Pittsburgh Sleep Quality Index (PSQI) and the Epworth Sleepiness Scale, visual analogue scales do not enable the differential diagnosis of isolated snoring and OSA, but allow the longitudinal documentation (e.g., after therapeutic intervention) [33, 34]. The Snore Outcome Survey (SOS), the Snoring Scale Score (SSS), and the Snoring Symptoms Inventory (SSI) represent other validated questionnaires for the assessment of the snoring intensity at baseline and also following therapeutic interventions [35–37], but are rarely used in clinical trials today. It must be emphasized that these questionnaires can only be answered by the bed partner, since the snoring patient is not able to assess the own snoring intensity.

Snoring is an acoustic phenomenon that can be described by objective parameters. The acoustic detection of snoring sounds should be done by air conduction (sound transmission through the air), since this is the only way to ensure that frequencies above 1000 Hz are adequately reproduced. The measurement of snoring sounds by body contact microphones or dynamic pressure measurement (sound transmission through the body), as it usually is the case in HST and PSG, leads to a reduction of the intensity spectra above 1000 Hz [38]. The acoustic analysis of snoring sounds, however, may contribute to the differential diagnosis and objective assessment of snoring in the near future, if the recording quality is standardized and improved. The scores usually provided in standard outpatient recording/polysomnography are currently not validated and can only be used to a limited extent for the qualitative or quantitative assessment of snoring, both intra- and inter-individually. Despite the positive data, the acoustic analysis of snoring sounds is therefore currently not suitable for routine diagnostics of isolated snoring. Pevernagie et al. have summarized suggestions for future research on the acoustics of snoring [39].

In conclusion, several gaps in the literature regarding the diagnosis and treatment of isolated snoring, and therefore areas for further research, have been identified. Future studies on these topics should pay attention to differentiate between isolated snoring and snoring in the context of obstructive sleep apnea with the help of sufficient objective testing. According to current standards, patients with an AHI above 5 events per hour should not be included in studies on isolated snoring. Another difficulty is the selection of the optimal endpoint in these studies. Usually, visual analogue scales are applied to evaluate the baseline snoring intensity and the effect of the particular treatment. No distinct objective evaluation method has gained general acceptance to date, even though the evaluation of the snoring intensity is usually done by the bed partner and therefore prone to multifactorial bias. With this regard, the authors (a) emphasize the particular need of randomized-controlled trials, since the majority of the existing literature is of low evidence, (b) indicate the reliable exclusion of OSA in studies on isolated snoring, and (c) encourage the application/establishment of validated outcome parameters.
